# Supplementary material for: The Development of Polylactic Acid/Multi-Wall Carbon Nanotubes/Polyethylene Glycol Scaffolds for Bone Tissue Regeneration Application
Source: Polymers (Basel). 2021 May 26;13(11):1740. doi: 10.3390/polym13111740 (PMC8198519; doi:10.3390/polym13111740)
Supplement: Supplementary file 1 [file polymers-13-01740-s001.zip › polymers-1223368-supplementary.pdf]

S.1 The preparation of polymer solutions and the corresponding ratios of MWCNTs and PEG to PLA in nanofibers

Table S1 The preparation of polymer solutions for electrospinning

	PLA	0.1C	0.5C	1.25C	3C	0.5C/0.1PEG	0.5C/1PEG	0.5C/10PEG
**Polymer solution A (in DCM)**	**10 g of 10% PLA**	**10 g of 10% PLA**	**10 g of 10% PLA**	**10 g of 10% PLA**	**10 g of 10% PLA**	**10 g of 10% PLA and 0.01% PEG**	**10 g of 10% PLA and 0.1% PEG**	**10 g of 10% PLA and 1% PEG**
**MWCNT dispersion B (in DMF)**	**6.25 g of pure DMF**	**6.25 g of 0.016 % MWCNT**	**6.25 g of 0.08 % MWCNT**	**6.25 g of 0.2 % MWCNT**	**6 g of 0.5 % MWCNT**	**6.25 g of 0.08 % MWCNT**	**6.25 g of 0.08 % MWCNT**	**6.25 g of 0.08 % MWCNT**
**Weight ratio of MWCNT to PLA in fibers**	**0%**	**0.1 %**	**0.5 %**	**1.25 %**	**3%**	**0.5 %**	**0.5 %**	**0.5 %**
**Weight ratio of PEG to PLA in fibers**	**0%**	**0%**	**0%**	**0%**	**0%**	**0.1%**	**1%**	**10%**

1. All percentages used in here are weight percentages
2. To fabricate DEX-loading nanofibers, 0.75 g of 4% DEX solution in DMF would be added to abovementioned electrospun solution so that the weight ratio of DEX to PLA would be 3%.

S2. The theoretical values of F/C molar ratio in DEX-loading nanofibers were determined by following calculation

The monomer of PLA (72g/mol) contains 3 carbons and no fluorine.

The monomer of PEG (44g/mol) contains 2 carbons and no fluorine.

DEX (392.46g/mol) contains 22 carbons and 1 fluorines.

I. **DL-PLA** (10 g of 10% PLA contains 0.75 g of 4 wt% DEX)

⇨ PLA=1g, DEX=0.03g in fibers

wt.% of C in fibers：

$$\frac{\frac{12*3}{72}+\frac{12*22}{392.46}*0.03}{1+0.03}*100\%=50.5\%$$

wt.% of F in fibers：

$$\frac{{}^{19}\!/_{392.46}*0.03}{1+0.03}*100\%=0.14\%$$

F/C mole ratio：

$$\frac{{}^{0.14}\!/_{19}}{{}^{50.5}\!/_{12}}*100\%=\mathbf{0.18}\%$$

II. **DL-0.5C** (10 g of 10% PLA contains 0.75 g of 4 wt% DEX, which was added to 6.25 g of 0.08%MWCNT)

⇨ PLA=1g, DEX=0.03g, MWCNT=0.005g in fibers

wt.% of C in fibers：

$$\frac{\frac{12*3}{72}+\frac{12*22}{392.46}*0.03+0.005}{1+0.03+0.005}*100\% = 50.7\%$$

wt.% of F in fibers：

$$\frac{{}^{19}/_{392.46}*0.03}{1+0.03+0.005}*100\% = 0.14\%$$

F/C mole ratio：

$$\frac{{}^{0.14}/_{19}}{{}^{50.7}/_{12}}*100\% = \mathbf{0.17}\%$$

III. DL-0.5C/0.1PEG (10 g of 10% PLA and 0.01% PEG contains 0.75 g of 4 wt% DEX, which was added to 6.25g of 0.08%MWCNT)

⇨ PLA=1g, PEG=0.001g, DEX=0.03g, MWCNT=0.005g in fibers

wt.% of C in fibers：

$$\frac{\frac{12*3}{72}+\frac{12*22}{392.46}*0.03+0.005+\frac{12*2}{44}*0.001}{1+0.03+0.005+0.001}*100\% = 50.74\%$$

wt.% of F in fibers：

$$\frac{{}^{19}/_{392.46} * 0.03}{1 + 0.03 + 0.005 + 0.001} * 100\% = 0.14\%$$

F/C mole ratio：

$$\frac{{}^{0.14}/_{19}}{{}^{50.7}/_{12}} * 100\% = \mathbf{0.17}\%$$

IV. DL-0.5C/1PEG (10 g of 10% PLA and 0. 1% PEG contains 0.75 g of 4 wt% DEX, which was added to 6.25g of 0.08%MWCNT)

⇨ PLA=1g, PEG=0.01g, DEX=0.03g, MWCNT=0.005g in fibers

wt.% of C in fibers：

$$\frac{\frac{12 * 3}{72} + \frac{22 * 12}{392.46} * 0.03 + 0.005 + \frac{12 * 2}{44} * 0.01}{1 + 0.03 + 0.005 + 0.01} * 100\% = 50.76\%$$

wt.% of F in fibers：

$$\frac{{}^{19}/_{392.46} * 0.03}{1 + 0.03 + 0.005 + 0.01} * 100\% = 0.14\%$$

F/C mole ratio：

$$\frac{0.14/_{19}}{50.76/_{12}} * 100\% = \mathbf{0.17}\%$$

V. **DL-0.5C/10PEG** (10 g of 10% PLA and 1% PEG contains 0.75 g of 4 wt% DEX, which was added to 6.25g of 0.08%MWCNT)

⇨ PLA=1g, PEG=0.1g, DEX=0.03g, MWCNT=0.005g in fibers

wt.% of C in fibers：

$$\frac{\frac{12 * 3}{72} + \frac{12 * 22}{392.46} * 0.03 + 0.005 + \frac{12 * 2}{44} * 0.1}{1 + 0.03 + 0.005 + 0.1} * 100\% = 51.06\%$$

wt.% of F in fibers：

$$\frac{19/_{392.46} * 0.03}{1 + 0.03 + 0.005 + 0.1} * 100\% = 0.13\%$$

F/C mole ratio：

$$\frac{0.13/_{19}}{50.76/_{12}} * 100\% = \mathbf{0.16}\%$$
